# A Rare Isolated Kidney Cyst: Hydatid Cyst

**DOI:** 10.7759/cureus.38566

**Published:** 2023-05-05

**Authors:** Emre Leventoğlu, Esat Burak Deniz, Nursel Kara Ulu, Meltem Polat, Kibriya Fidan

**Affiliations:** 1 Department of Pediatric Nephrology, Gazi University, Ankara, TUR; 2 Department of Pediatrics, Gazi University, Ankara, TUR; 3 Department of Pediatric Infectious Disease, Gazi University, Ankara, TUR

**Keywords:** computerized tomography, kidney ultrasound, general pediatrics, parasitic disease, benign renal mass

## Abstract

Hydatid disease is a parasitic disease caused by *Echinococcus granulosus* or *Echinococcus multilocularis*. It is still a serious public health problem in endemic regions such as the Mediterranean basin. Since the complaints caused by the cysts are non-specific and routine laboratory tests do not always yield positive results, diagnosis may be difficult. While liver involvement is present in 70% of cases, larvae escaping from the filtration of the liver cause pulmonary disease in 25% of cases. Although the prevalence of kidney involvement in all hydatid cysts is approximately 2-4%, and isolated kidney involvement is extremely rare at 1.9%. In this case report, we present an extremely rare pediatric case of isolated renal hydatid cyst, the diagnosis of which was somewhat delayed.

## Introduction

Hydatid disease is a parasitic disease caused by *Echinococcus granulosus* and rarely by *Echinococcus multilocularis* called alveolar disease. Hydatid cyst infection is still a serious public health problem in endemic regions such as the Mediterranean basin. Since the complaints caused by the cysts are non-specific and routine laboratory tests do not always yield positive results, diagnosis may be difficult [1]. While liver involvement is present in 70% of cases, larvae escaping from the filtration of the liver cause pulmonary disease in 25% of cases. Although the prevalence of kidney involvement in all hydatid cysts is approximately 2-4%, and isolated kidney involvement is extremely rare at 1.9% in the general population [2,3]. In this case report, we present an extremely rare pediatric case of an isolated kidney hydatid cyst, the diagnosis of which was somewhat delayed.

## Case presentation

**Figure 1:**
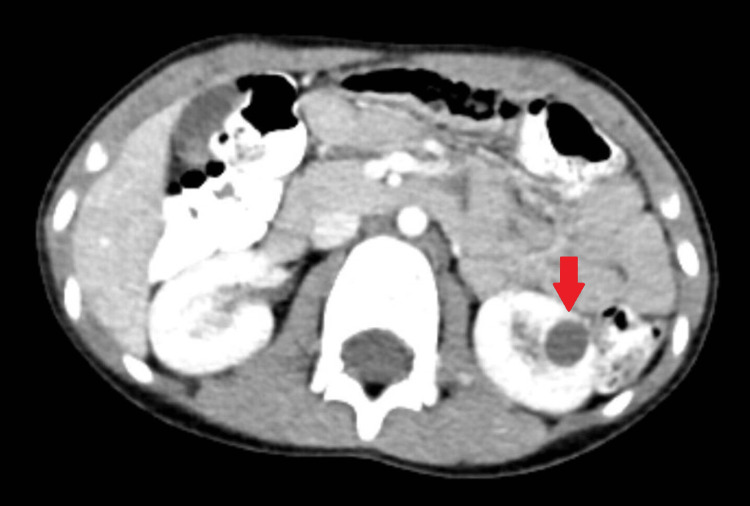
Abdominal tomography image of the patient The arrow points to an 18x17 mm cystic lesion with lobulated contour and membranous structures in the lower-central part of the left kidney.

A 10-year-old male patient was referred to our hospital from a health institution because of left flank pain, and an abdomen ultrasound revealed a 13 mm cortical cyst in the lower pole of the left kidney. No positive findings were found on physical examination and laboratory evaluation. Therefore, control imaging with ultrasound was planned. Six months later, abdominal tomography was performed because our patient had a history of blunt trauma due to an accident. The tomography showed an 18x17 mm cystic lesion with lobulated contour and membranous structures in the lower-central part of the left kidney (Figure 1).

As a result of the evaluation of radiological findings, an indirect hemagglutination test (IHA) was performed, considering that the kidney mass might be a hydatid cyst, and the test result was negative (≤1/160). However, during this period, urticarial plaques developed on the face and trunk and eosinophilia (8.9%) was detected on the hemogram. As the patient occasionally visited relatives in a village and the history of contact with cats and dogs in that village was learned, the IHA test for hydatid cyst was performed again in a private center and the result was positive. Radiological examinations revealed no other lesion in the thorax and abdomen except for the cyst in the kidney. Oral albendazole at a dose of 10 mg/kg/day was started prophylactically for three weeks preoperatively. The patient was then explored by an extraperitoneal approach under general anesthesia, and a cystectomy was performed. The patient had no intraoperative or postoperative problems. Albendazole treatment was continued for another six months postoperatively.

## Discussion

Hydatid disease is a zoonotic disease. The definitive hosts of hydatid disease are canids such as dogs, wolves, foxes, and coyotes. Humans are incidental hosts, and the disease can affect almost any system [4]. While kidney involvement may be associated with systemic involvement in hydatid cysts, isolated kidney hydatid cysts are rare [5]. The cyst grows slowly in the kidney, usually over 5-10 years, with an average age at diagnosis of 30 years. Therefore, the diagnosis of renal hydatid cysts in the pediatric age range is much rarer [6]. The clinical presentation of kidney hydatid cysts can range from asymptomatic to loss of kidney function. The most common complaint is non-specific flank pain due to chronic compression of the cyst, as in our patient. Hematuria and hydatiduria may also accompany the complaint of flank pain due to the opening of the cyst into the collecting system [7]. Hydatiduria is a pathognomonic sign of hydatid cysts of the kidney, however, this finding is present in only 10-20% of cases [8]. Since it is a parasitic infection, allergic reactions may be seen in patients, as in our patient [9]. Considering the rarity of the disease, hydatid cysts should be considered in the differential diagnosis of non-specific flank pain, hematuria, and allergic reactions. Although IHA is a sensitive test, false negative results may be observed. Positive serologic tests confirm the diagnosis, while negative tests do not rule out it [10]. Hydatid cysts can be visualized and evaluated by ultrasonography, computed tomography, or magnetic resonance imaging. Ultrasonography is the most widely used modality. However, computed tomography or magnetic resonance imaging may be useful for situations where more anatomical detail is needed [11]. Among imaging modalities, it shows well-circumscribed unilocular or multilocular thick-walled cysts with prominent walls. The detection of daughter vesicles on ultrasound or tomography is also characteristic of hydatid cysts [7].

Treatment of hydatid cysts usually involves antiparasitic therapy combined with either surgical resection of the cyst or percutaneous management [12]. For cysts smaller than 5 mm, albendazole used alone at a dose of 10-15 mg/kg/day is sufficient, while combined treatments are necessary for cysts >5 mm in diameter, as in our patient [13]. The optimal duration of definitive and adjunctive drug treatment is uncertain. Drug therapy for definitive treatment usually consists of one to six months. Drug therapy for adjunctive treatment consists of a few weeks before surgery and at least one month after surgery [14]. In our patient, albendazole was used for three weeks preoperatively and six months postoperatively.

Hydatid cysts can relapse years after treatment. The optimal approach for monitoring is uncertain and should be individualized according to patient characteristics and available resources [15].

## Conclusions

Kidney hydatid cysts resemble slow-growing masses. Late diagnosis due to vague clinical manifestations is the main cause of loss of kidney function. In endemic areas, it is useful to consider kidney hydatid cysts in patients with non-specific urinary tract and flank pain, even if the IHA test is negative. Awareness of this parasitic disease needs to be raised and further studies should be conducted to establish a treatment protocol.
